# Exogenous application of silicon improves the performance of wheat under terminal heat stress by triggering physio-biochemical mechanisms

**DOI:** 10.1038/s41598-021-02594-4

**Published:** 2021-11-30

**Authors:** Talha Mustafa, Abdul Sattar, Ahmad Sher, Sami Ul-Allah, Muhammad Ijaz, Muhammad Irfan, Madiha Butt, Mumtaz Cheema

**Affiliations:** 1 College of Agriculture, Bahauddin Zakariya University, Bahadur Sub-Campus Layyah, Layyah, Pakistan; 2 Department of Agronomy, Bahauddin Zakariya University, Multan, Pakistan; 3 School of Science and the Environment, Grenfell Campus, Memorial University of Newfoundland, Corner Brook, NL A2H 5G4 Canada

**Keywords:** Plant sciences, Plant stress responses, Abiotic, Heat

## Abstract

Due to climate change, temperature in late February and early March raised up which cause heat stress at reproductive stage (terminal growth phase of wheat crop) which has become the major causative factor towards low wheat production in arid and semiarid regions. Therefore; strategies need to be adopted for improving terminal heat stress tolerance in wheat. In this study, we assessed whether foliar application of silicon (Si) (2 and 4 mM) at terminal growth phase i.e. heading stage of wheat imposed to heat stress (37 ± 2 °C) under polythene tunnel could improve the performance of wheat. Results of the study revealed that heat stress significantly reduced the photosynthetic pigments (chlorophyll a, b and a + b and carotenoids) leading to a lower grain yield. However, a 4 mM Si application (foliar applied) at heading stage prominently increased the chlorophyll a, b and a + b and carotenoids of flag leaf by improving the activities of enzymatic antioxidants (catalase, peroxidase and superoxide dismutase) and osmoprotectants (soluble sugar protein and proline) under terminal heat stress. Improvements in the performance of wheat (chlorophyll contents, carotenoids, soluble sugar and proteins and proline and yield) with foliar application of Si were also observed under control conditions. Correlation analysis revealed strong association (*r* > 0.90) of chlorophyll contents and carotenoids with grain and biological yield. Negative correlation (−0.81 < *r* > −0.63) of physio-biochemical components (antioxidants, proline, soluble sugars and proteins) with yield revealed that under heat stress these components produced in more quantities to alleviate the effects of heat, and Si application also improved these physio biochemical components. In crux, foliar application of Si alleviates the losses in the performance of wheat caused by terminal heat stress by improving the antioxidant mechanism and production of osmoprotectants.

## Introduction

Temperature of the globe is increasing due to climate change which is also affecting crop phenology^1^. Wheat is an important cereal crop of the globe and sensitive to terminal heat stress imposed at reproductive stage^2,3^. It has been observed from couple of decades that temperature in late February has been raised than the normal^4,5^ which has become one of the key growths limiting factors during the reproductive stage as it reduces metabolism and photosynthetic partitioning and pollen viability^1,6^. High ambient temperature at pollination and grain filling phases is most harmful to plants; referred to as terminal heat stress, which disturbs metabolic activities^3^.

Stomatal conductance and photosynthetic processes are badly affected by increased amount of (ROS) in different cellular organelles^7^ in response to the heat stress, resulting in oxidative damage^8^. Oxidative damage through production of reactive oxygen species (ROS) is resulted due to elevated temperatures, mainly in chloroplasts. Because of high temperature, chlorophyll molecules undergo over-excitation that results in production of ROS. Plants are prepared for ROS scavenging in stressed circumstances with an internal defense mechanism manifested with catalase (CAT), peroxidase (POX) and superoxide dismutase-SOD^9,10^. In addition, heat stress reduces the duration of grain filling, cause oxidative damage to the photosynthetic apparatus and reduces the dry matter accumulation in grains resulting in a lower grain yield of wheat^11–13^. Heat stress during grain filling reduces the sink capacity of wheat, thus by affecting hexoses transport across the plasma membrane^14^ and results in lower accumulation of assimilates in the grains and thus results in shorter and shrinked grains^15^.

Exogenous use of mineral elements, organic and in organic substances plays a critical role in improving the growth and production of plants as well as in mitigating the effect of abiotic stress and, as a result, improving economic yields^16^. Several studies have shown the importance of micronutrients in offering resistance to plants against different stresses^17^. Silicon (Si) is one of the mineral elements that is attracting attention due to its essential role in enhancing biotic and abiotic stress tolerance. The Si, taken up by the roots, travels through transpiration streams to aerial of plants^18^. In addition, through control of osmo-protectant, antioxidant, and secondary metabolites, Si mitigates the harmful effects of abiotic stress^19,20^. In early seedlings, Si has been documented to induce heat stress tolerance in *Salvia splendens*^21^, rice metal tolerance^22^, tomato salinity tolerance^23^. Maghsoudi et al.^24^ reported that the photosynthetic pigments in wheat plants were increased by foliar Si application. The alleviating role of Si for heat stress tolerance may be attributed to increase Si in leaf and regulates transpiration and uptake of other micro and macro nutrients leading to cell homeostasis^25^. Si, either applied in the form of foliar or nano particles, restores heat stress damages in wheat cell organelles especially nucleus and chloroplast and thus improves photosynthetic efficiency resulting in more production of assimilates and ultimately more biomass^26,27^. Si provides mechanical strength to the stem by increasing the lignin content which make it withstand harsh environmental conditions^28^. Saha et al.^29^, reviewed the role of Si in heat stress tolerance and reported direct relationship of Si concentration in leaf tissue and heat stress tolerance. They further reported that Si alleviates heat stress by improving photosynthesis, membrane stability, water balance and by enhancing the expression of heat stress responsive genes.

Concluding the discussion, role of Si in agriculture has been well explored, but in case of wheat most of the studies explore its effects on seedling stage or drought stress, effect of Si on alleviation of terminal heat stress in wheat have often been ignored. Furthermore, information regarding the regulatory functions of Si in ROS metabolism under terminal heat stress by activating antioxidant defense mechanism in wheat is also limited. Considering the above facts, major objectives of the study were to explore the potential of Si in ameliorating the adversities of terminal heat stress through morphological, physiological and antioxidant defense mechanisms in wheat. In addition, to evaluate the effect of the silicon on the yield and yield characteristics of terminal heat stress. The tested approach can therefore be revealed as an effective for wheat management that can be readily adopted by farmers to avoid the detrimental effects of terminal heat stress and increase wheat productivity to ensure global food security.

## Materials and methods

### Experimental site description

Pot experiment was conducted in the greenhouse at College of Agriculture, BZU Bahadur Campus Layyah, Pakistan (longitude 70° 56′ 20.5" E, latitude 30° 57′ 40.6" N, and altitude 151 m) to study the potential of Si to mitigate the adverse effect of terminal heat stress on bread wheat. Wheat variety Anaj-17 (developed by Ayub Agriculture Research Institute) obtained from Punjab Seed Corporation, Pakistan that is a commonly cultivated variety of wheat in that region was used in the experiment. It has high-yield, stability, and adaptability and is widely grown in Layyah district of Pakistan. Ten seeds were sown in each pot (90 cm in height, 20 cm in diameter) containing 15 kg sandy loam soil. After one week of emergence five plants per pot were maintained for the subsequent studies.

### Crop husbandry

The nitrogen (N), phosphorus (P) and potassium (K) fertilizers were applied at the rates of 45, 25 and 30 mg kg^-1^ of soil to sustain the emergence of wheat seedlings. The sources of fertilizers applied were urea, di-ammonium phosphate and potassium sulphate. All the fertilizers were thoroughly mixed at the time sowing. Irrigation was applied with normal interval to avoid any drought stress during the entire experiment period. The soil used in experiment was sandy loam with pH 8.5, electrical conductivity (EC) 2.56 dSm^-1^, organic matter 0.76%, total nitrogen 0.58 g kg^-1^, available phosphorous of 9.53 mg kg^-1^, and available potassium of 62.34 mg kg^-1^, bulk density of 1.71 g cm^-3^.

### Experimental design and treatments

Experiment was laid out in Completely Randomized Design (CRD) with two factors factorial arrangements. One factor was temperature regimes: ambient temperature (control) and heat stress (polythene tunnel). Second factor was various levels of silicon application: 0 (control), water spray, 2 and 4 mM Si. There were 24 treatments in total (2 temperature regimes × 4 Si levels × 4 replicates). There were four replicates per treatment and each replicate consisted of two pots with five plants per pot. Thus, there were 40 plants in 10 pots for each treatment. At heading stage (BBCH-55), pots were divided into two groups. One group was placed under polythene sheet (heat stress). The pots of other group were kept in open normal condition served as control (ambient temperature) till reached to harvesting phase. One week (7 days) after the imposition of heat stress, Si was applied including 2% tween-20 as a surfactant. In both the pot groups, one set of plants was unsprayed and the other was sprayed with distilled water (controls).

### Imposition of heat stress

**Figure 1:**
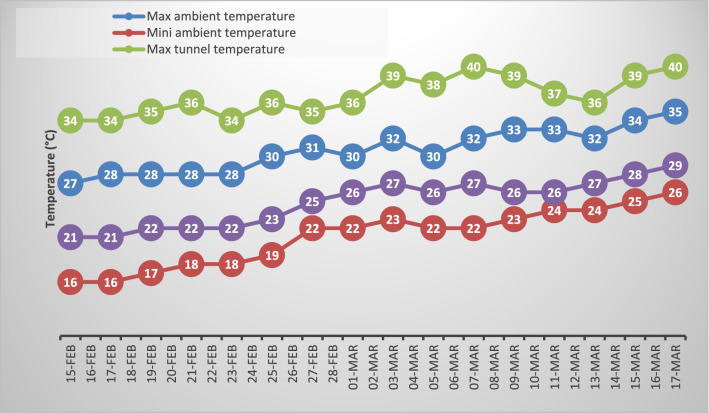
Minimum and maximum temperature recorded outside (ambient temperature) and inside (heat stress) the plastic tunnel when the wheat plants reached to heading stage (75 days after sowing) for 30 days.

**Figure 2:**
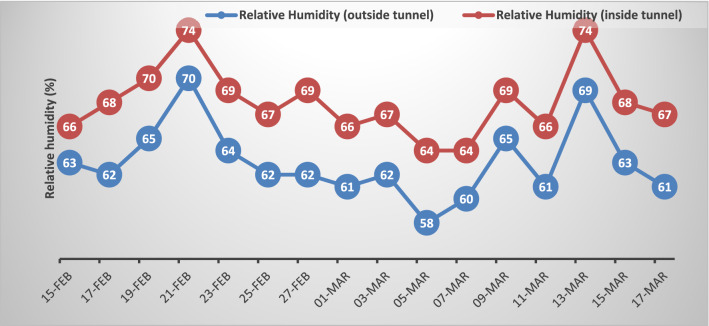
Relative humidity recorded outside and inside the plastic tunnel when the wheat plants reached to heading stage (75 days after sowing) for 30 days.

Heat stress was imposed during BBCH-55 growth stage of wheat. A plastic tunnel made of transparent polythene sheet was made above the pots by using bamboo sticks. Tiny holes were made in polythene sheet to minimize the humidity. The pots in control treatment were placed under normal conditions. Humidity probe (Digital Multimeter-50302) and Digital temperature was used to note temperature and humidity. During heat stress, the temperature of control and heat-stressed pots was recorded twice a day and averaged. Temperature was 5–8 °C higher inside the polythene sheet than ambient condition during daytime. Minimum and maximum temperature data inside the polythene sheet tunnel and open air ambient condition were recorded at alternate interval (Figs. 1, 2).

### Plant sampling for biochemical analyses

Plant leaves samplings were done after 7 days of Si application and 14 days after the imposition of heat stress to measure chlorophyll and carotenoid contents, osmolytes determination (the total soluble sugars, protein and free proline) and enzymatic antioxidants activities. Healthy full expanded and undamaged flag leaf of wheat plant was taken from all experimental units. After cleaning, the leaves of wheat plants were frozen with liquid N_2_ immediately and stored at −80 °C for biochemical analyses. While all other attributes like yield and its components were recorded at harvest.

### Chlorophyll and carotenoids

Keeping in view the Arnon’s procedure^30^, 0.5 g fresh fully expanded flag leaves were taken. At 0–4 °C, 80% of 5 mL acetone was used for extraction, overnight. The supernatant was separated after centrifugation (10,000×*g* for 5 min) for absorbance reading at 645 and 663 nm for the chlorophyll a, and chlorophyll b respectively by using the Spectrophotometer (Hitachi-U2001, Tokyo, Japan). However, for the determination of carotenoid absorbance reading was recorded at at 480 nm as described by Arnon^30^.

### Osmolytes determination

Healthy fresh green flag leaves (0.5 g) were taken to determine the total soluble sugars, protein and free proline. Each sample was grounded using 1 mL extraction buffer (pH 7.2) in a pre-chilled mortar pestle. Before extracting the proteins from the samples, cocktail protease inhibitors having 1 µM concentration was added in the saline phosphate buffer containing the 2 mM KH_2_ PO_4_,2.7 mM KCl, 10 mM Na_2_HPO_4_ and 1.37 mM NaCl dissolved in 1 L of di-ionized H_2_O. HCl was used to adjust buffer pH and autoclaved. Centrifugation of extracted samples was done at 12,000×g for 5 min. Pellet was discarded, and supernatant was stored in centrifuge tube for measuring the quantity of soluble proteins. Bradford^31^ assay was followed for the amount total soluble proteins determination. To construct standard curves, various dilutions (10, 20, 30, 40, 50, 60, 70,80, 90 and 100 µg µL^-1^) of Bovine serum albumin were used. Tubes were incubated and vortexed at room temperature up to 30 min, after addition of the 400 mL µL Dye stock and DI water. UV 4000 UV–VIS spectrophotometer was used to record absorbance of the samples. Proline was determined as described by Simaei et al.^32^ Using10 mL of sulpho salicylic acid (3% w/v), fresh leaf samples of 0.5 g were filtered and homogenized. For color development, firstly, filtrate was taken in test tubes. Then it was treated with glacial acetic acid and ninhidrine (2.5%). After that these were retained in water bath whose temperature was elevated to 100 °C for period of 60 min. After exclusion from water bath, toluene was added to test tubes for chromophores separation. Using UV–VIS spectrophotometer, optical density (520 nm) was measured. Following the procedure of Giannakoula et al.^33^, soluble sugar contents were measured in this extract.

### Enzymatic antioxidants activities

For extraction of enzymatic antioxidants from centrifuged fresh leaf sample (15,000×g for 20 min), 5 ml of phosphate buffer (50 mM with 7.8pH) was used. As result of photochemical reduction, superoxide dismutase (SOD) activity was calculated through the prevention of nitroblue tetrazolium (NBT) at 560 nm^34^. The reactants of the reaction were 1 mL NBT (50 µM), 1 mL riboflavin (1.3 µM), 50 µL enzyme extract, 950 µL phosphate buffer (50 mM), 500 µL methionine (13 mM) and 500 µL EDTA (75 mM). This process was initiated by holding reaction mixture under illuminations of 30 W fluorescent lamp. After 5 min of lamp turned off, the reaction was stopped. At 560 nm blue formazane formation was observed that was resulted due to NBT photo reduction. Using same reactants but having no enzyme extract, blank reading was taken. By measuring the change in the absorbance due to H_2_O_2_ produced as a result of enzyme reaction, catalase (CAT) activity was recorded at 240 nm using a UV–visible spectrophotometer. To initiate the reaction, the reaction mixture (900 µL H_2_O_2_ (5.9 mM) and 2 mL phosphate buffer (50 mM) was added with 100 µL enzyme extract. µmol of H_2_O_2_ per minute per mg of protein was used to define catalase^35^. The peroxidase (POD) activity was estimated following the procedure of Kar and Mishra^36^. The reactants used were composed of 5 ml of Tris–HCL buffer (0.1 M), 5 ml pyrogallol (10 mM), 5 mM of H_2_O_2_ (5 mM) and 100 µL enzyme extract. The By noting the decline in the absorbance at 425 nm which was due to H_2_O_2_ dependent oxidation of pyrogallol, POD activity was measured. This was further expressed as POD IU per minute per mg of the protein.

### Yield and yield-related traits

At maturity, plant height and spike length of individually selected plant from each pot was measured from soil surface to the tip of the ear, with the help of meter rod. From each pot number of fertile tillers per plant was counted at maturity. Plants were harvested and threshed manually to record the number of spikelets per spike, number of grains per spike, 100 grain weight (g), and grain yield per plant (g). Harvest index (HI) was calculated by the following formula as given by Hunt^37^:

$${\rm H.I}. = \frac{{{\text{Economic}}\;{\text{yield}}}}{{{\text{Biological}}\;{\text{yield}}}} \times 100$$

### Statistical analysis

Using Fisher’s Analysis of Variance technique, all the data of the experiment was analyzed and average of treatments was computed by LSD test^38^ at 5% probability level. Pearson linear correlation was run to assess the association among different traits. Figures were prepared using Microsoft Excel 365.

### Compliance with the regulation

The study complies with local and national regulations.

### Permissions

For the collection of seeds/plants, all relevant permits or permissions have been obtained where applicable.

## Results

### Chlorophyll and carotenoids

**Figure 3:**
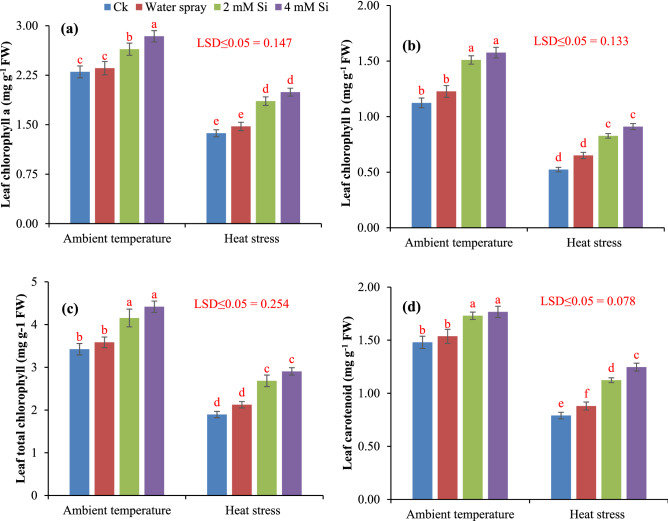
Influence of exogenously applied Si on chlorophyll *a* (**a**), chlorophyll *b* (**b**), total chlorophyll (**c**) and carotenoid (**d**) of wheat under ambient temperature and heat stress. Values are the mean SE of four replicates.

Wheat plants subjected to foliar Si application under both ambient temperature and heat stress condition upgraded the chlorophyll a, b and a + b and carotenoids contents at 2 mM and 4 mM concentration. Changes in chlorophyll and carotenoids due to heat stress and Si application are presented in Fig. 3. Application of 2 mM Si significantly improved the chlorophyll a (14.7%), chlorophyll b (34.82%), chlorophyll a + b (21.34%) and carotenoids (16.89%) in ambient conditions as compared to control (ck). Likewise, application of 4 mM Si significantly improved the chlorophyll a (23.47%), chlorophyll b (41.07%), chlorophyll a + b (29.23%) and carotenoids (19.59%) in ambient conditions as compared to control (ck). However, in heat stress condition 2 mM Si application significantly improved the chlorophyll a (35.76%), chlorophyll b (59.61%), chlorophyll a + b (41.79%) and carotenoids (41.77%) and 4 mM Si application significantly improved the chlorophyll a (45.25%), chlorophyll b (75%), chlorophyll a + b (53.43%) and carotenoids (58.22%) compared with control.

### Enzymatic antioxidants

**Figure 4:**
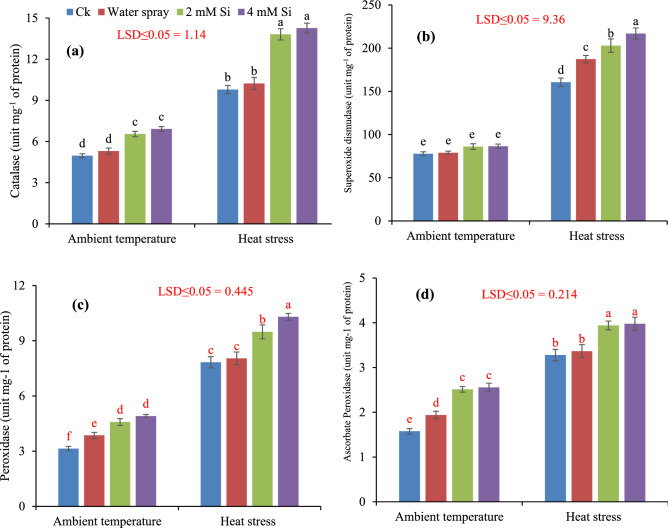
Influence of exogenously applied Si on catalase (**a**), superoxidase dismutase (**b**), peroxidase (**c**) and ascorbate peroxidase (**d**) of wheat under ambient temperature and heat stress. Values are the mean SE of four replicates.

The results show that activity of enzymatic antioxidants in flag leaves of wheat was higher in heat stress condition when compared to ambient temperature. However, Si application significantly increased the enzymatic antioxidants activity. Changes in chlorophyll and carotenoids due to heat stress and Si application are presented in Fig. 4. According to results, in heat stress condition, CAT activity was increased 41.06 and 45.76% with 2 mM and 4 mM Si application. The SOD activity of plant leaves was also higher under heat stress as compared to ambient temperature. Under ambient temperature, SOD activity was lower in control (Ck) while foliar application of Si increased 10.69 and 11.19% SOD activity. Similarly, under heat stress, 2 mM and 4 mM Si application enhanced 26.40 and 35.12% SOD activity respectively as compared to control (Ck) treatment. Compared with ambient temperature, POD activity was higher in heat stress condition while 2 mM and 4 mM foliar application of Si increased 21.2 and 31.54% POD activity respectively when compared to non-treated plants. While in ambient temperature, 2 mM and 4 mM enhanced POD activity to 45.85 and 56.36% respectively. The activity of APX was higher in heat stress conditions as compared to ambient temperature. At heat stress, 4 mM Si increased 21.34% APX.

### Osmo-protectants

**Figure 5:**
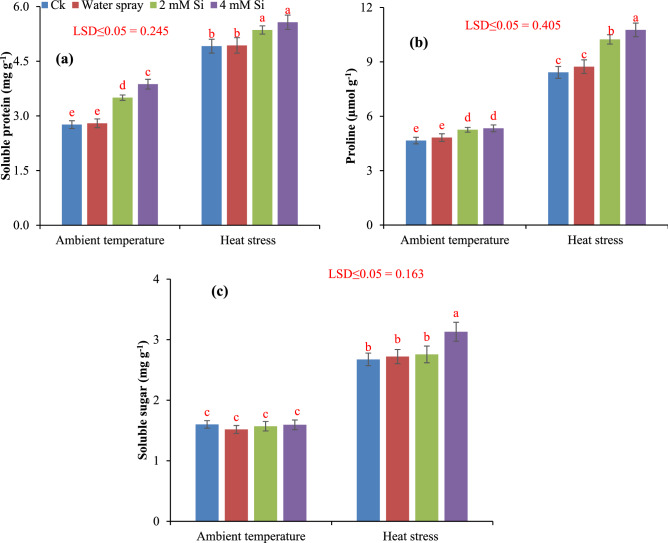
Influence of exogenously applied Si on soluble protein (**a**), proline (**b**) and soluble sugar (**c**) of wheat under ambient temperature and heat stress. Values are the mean SE of four replicates.

Soluble protein, proline and sugar contents in wheat leaves were significantly increased by exogenously applied silicon under both ambient and heat stress conditions. Changes in osmo-protectants due to heat stress and Si application are presented in Fig. 5. The increased 26.81% and 40.21% protein content in ambient condition and 9.16% and 13.44% under heat stress. Similarly, proline concentrations were increased (21.6% and 27.8%) in flag leaves of wheat by application of 2 mM and 4 mM Si under heat stressed plants. While application of Si at the rate of 4 mM significantly enhanced the soluble sugar content by 17.22% in heat stress condition while Si didn’t showed any significant effect on sugar content under ambient condition.

### Yield and yield components

**Figure 6:**
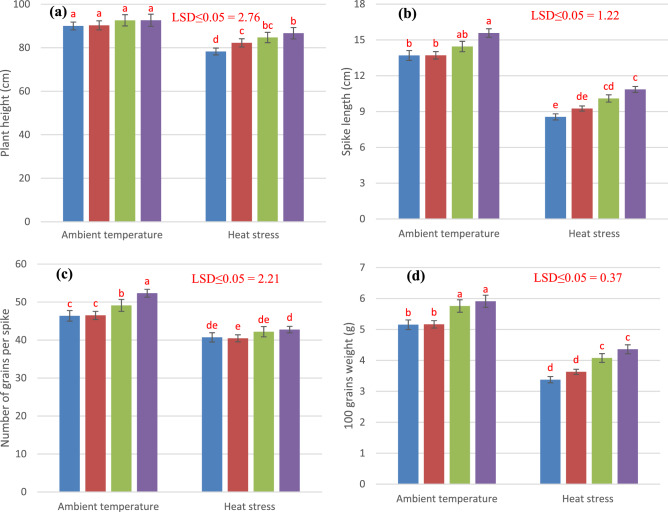
Influence of exogenously applied Si on plant height (**a**), spike length (**b**), number of grains per spike (**c**) and 100 grains weight (**d**) of wheat under ambient temperature and heat stress. Values are the mean SE of four replicates.

**Figure 7:**
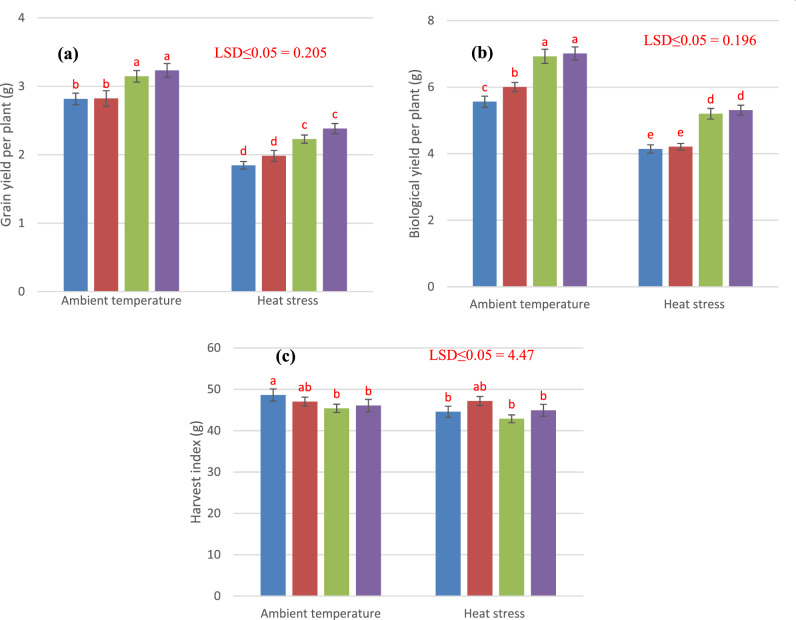
Influence of exogenously applied Si on grain yield (**a**), biological yield (**b**) and harvest index (**c**) of wheat under ambient temperature and heat stress. Values are the mean SE of four replicates.

The results show that plant height was significantly reduced by heat stress while increased significantly by foliar application of Si under heat stress. Changes in chlorophyll and carotenoids due to heat stress and Si application are presented in Figs. 6 and 7. In ambient temperature, Si application shows the non-significant response in comparison to control treatment. In comparison to control treatment, 2 and 4 mM Si application increased 8.24 and 10.7% plant height correspondingly under heat stress. Terminal heat stress significantly reduced the spike length, but foliar application of Si significantly increased the spike length of wheat under ambient temperature as well as under heat stress. Foliar applied 2 mM and 4 mM Si improved 18.1 to 26.9% in spike length under heat stress. Number of grains per spike was reduced significantly by the imposition of terminal heat stress. Whereas; foliar application of Si enhanced number of grains per spike under normal and heat stress conditions. Under ambient condition, 4 mM Si application increased 12.8% number of grains per spike while, 5.06% increase was observed under heat stress as compared to control treatment. Significant reductionin100-grains weight under heat stress was observed as compared to ambient temperature. However, 4 mM foliage applied Si increased 14.75 and 28.9% 100-grains weight under heat stress and ambient temperature respectively that was statistically at par with 2 mM Si application. Under heat stress foliage applied 2 mM and 4 mM Si increased 25.6% and 28.26% biological yield per plant. Likewise, at ambient temperature, Si application at the rate of 2 mM and 4 mM enhanced 24.64% and 26.07% biological yield when compared to control treatment where Si was not applied. Grain yield per plant was reduced significantly under heat stress as compared to ambient temperature but foliar application of Si increased grain yield per plant under both ambient temperature and heat stress conditions. At ambient temperature 2 mM and 4 mM Si increased grain yield 11.70% and 14.53% respectively. While under heat stress Si application at the rate of 2 mM and 4 mM increased grain yield to 20.54% and 28.64% respectively. According to results heat stress lowered the harvest index of wheat crop. While under heat stress Si applied at the rate of 4 mM increased harvest index to 0.76%.

### Correlation analyses

Correlation analyses revealed a strong association among different physiological, biochemical and yield related traits. Morphological traits (plant height, number of grains per spike, spike length) and leaf pigments (chlorophyll and carotenoids) showed strong and positive association with grain yield and biological yield which range from 0.91 to 0.96. Antioxidant enzymes and osmo-protectant are produced in more quantities in response to the external stress. Therefore, they showed a negative correlation with yield components. Their correlation ranged – 0.63 to – 0.81. Moreover, a strong association was observed among different antioxidant enzymatic activities and among different osmo-protectants (Table 1).

**Table 1:** Correlation analyses among different yield related traits, physiological traits, antioxidant enzymatic activities and osmo-protectant.

	PH	SL	NGPS	GYP	BYP	CAT	SOD	POD	APX	A + B	CARO	PROT	PROL	SUG
PH	1.00													
SL	**0.92**	1.00												
NGPS	**0.83**	**0.89**	1.00											
GYP	**0.92**	**0.94**	**0.93**	1.00										
BYP	**0.91**	**0.91**	**0.93**	**0.95**	1.00									
CAT	**– 0.55**	**– 0.68**	**– 0.62**	**– 0.63**	– 0.49	1.00								
SOD	**– 0.68**	**– 0.81**	**– 0.76**	**– 0.77**	**– 0.67**	**0.95**	1.00							
POD	**– 0.63**	**– 0.76**	**– 0.69**	**– 0.71**	**– 0.58**	**0.97**	**0.98**	1.00						
APX	**– 0.58**	**– 0.70**	**– 0.59**	**– 0.63**	– 0.48	**0.95**	**0.94**	**0.98**	1.00					
A + B	**0.92**	**0.96**	**0.93**	**0.96**	**0.94**	**– 0.58**	**– 0.74**	**– 0.66**	**– 0.58**	1.00				
CARO	**0.95**	**0.96**	**0.90**	**0.95**	**0.93**	**– 0.59**	**– 0.75**	**– 0.68**	**– 0.61**	**0.92**	1.00			
PROT	**– 0.65**	**– 0.76**	**– 0.63**	**– 0.69**	**– 0.56**	**0.94**	**0.95**	**0.92**	**0.95**	**– 0.64**	**– 0.68**	1.00		
PROL	**– 0.67**	**– 0.79**	**– 0.73**	**– 0.74**	**– 0.63**	**0.94**	**0.94**	**0.94**	**0.95**	**– 0.70**	**– 0.71**	**0.95**	1.00	
SUG	**– 0.73**	**– 0.84**	**– 0.80**	**-0.81**	**– 0.73**	**0.92**	**0.92**	**0.96**	**0.90**	**– 0.79**	**– 0.80**	**0.93**	**0.94**	1.00

For bold values p < 0.01; for non-bold values p < 0.05; n = 24.

## Discussion

Under field condition crops are exposed to various stresses (biotic and abiotic) that which significantly restricted plants growth by limiting yield and productivity^39–41^. In current study, the heat stress hampered plants morpho-physiological and antioxidants attributes and decreased the grains yield of wheat; nevertheless, addition of silicon (Si) was more favorable in reducing the adversarial exposure of heat and temperature stress. Numerous studies have highlighted the improvement in plant growth and development with Si addition under different abiotic stress in many crop species i.e. rice^42^, wheat^43^, soybean^44^, and sorghum^45^ due to positive impact of Si on plants mechanical strength and minerals nutrition, ultimately plants resistance to abiotic stress. In present study, with increasing temperature regimes the chlorophyll a, chlorophyll b, and total carotenoid contents were gradually decreased. Rossi et al.^46^ reported that under heat stress activities of chlorophyll degrading peroxidase and chlorophyllase increased in bent grass (*Agrostis *spp.) which is main cause of lower chlorophyll pigments. Changes in chlorophyll a and b were similar due to heat stress and Si improved both components in a same magnitude, especially under heat stress. Chovancek et al.^47^ showed that recovery of photosystem I, from heat stress is poor in hexaploidy wheat than tetraploid wheat due to higher values of electric membrane potential. Si protect the damage of chlorophyll pigments and photosystem I and carotenoids by enhancing the production of antioxidants and osmoprotectants^48,49^ (Figs. 4, 5) and thus reduced the photosynthetic injury under heat stress.

The heat stress induced the significant modulation in antioxidant enzymatic POD, SOD, CAT and APX activities in wheat. Results are in agreement with Hussain et al.^50^ documented that addition of Si induced marked variation in SOD, POD, and CAT in two barley cultivars under different regimes. SOD detoxify highly toxic O_2_^-^ into H_2_O_2_^51^. CAT and APX is an important enzyme of ascorbate–glutathione metabolism that scavenge different ROS especially H_2_O_2_^52,53^. The silicon reported to scavenge ROS production by improving the antioxidant enzymatic activities^54,55^. The increase in POD activity helps the plants to circumvent oxidative destruction^51^. So, addition of Si may enhance the POD activity signifying in biosynthesis of lignin and suberin that build-up a physical obstacle against stress^56^. The activity of CAT increased with increasing stress which has been stated in maize^53^ and rice^56^. Many studies have documented to decrease oxidative destruction by increasing enzymatic activities in chickpea^57^. and sunflower^58^ under drought stress. Li et al.^59^ stated that application of Si increased the chlorophyll content, net photosynthetic rate, SOD, POD, CAT, and APX and restrained permeability leaf plasma membrane. Silicon assists the plant to retain and take up maximum water to improve the water status of the upper parts of plants. The silicon facilitated amelioration of oxidative damage under stress condition is incompatible with enhancement of antioxidative defense (proline, glutathione, catalase, peroxidase, superoxidase dismutase, calcium, potassium, silicon, and ascorbic acid). Moreover, in plants the accumulation and of proline is the sign of osmotic stress response because it is essential osmolyte and play significant role to adjust osmotic potential in plants cell^60^. In present study with application of Si significantly improved the proline, soluble sugar and soluble protein content in wheat. While these increased accumulations were vibrant with Si 4 mM for temperature stress along with heat stress environment. Exogenous application of silicon increased the plants tolerance to the stress. Furthermore, glutathione, ascorbic acid, and soluble sugar content were increased with addition of silicon^60^. The accretion of organic solutes particularly soluble sugars contents are the basic solutes that were involved in osmotic potential adjustment in glycophytic plants under osmotic stress condition^61^. The improvement in antioxidants and osmolytes with addition of Si is an effective mechanism to enhance the plants tolerance to oxidative stress. The application of Si 4 mM significantly increased the soluble protein, sugars and proline content under heat stress. The accumulation of protein in response to silicon may be due to important role of Si in specific protein synthesis, functioning of mRNA^62^ and DNA formation^63^. Improvement in physiological and biochemical attributes due to Si lead to a better yield under heat stress condition. Si provides dehydration tolerance to the plants at cellular level and facilitates the assimilation of photosynthates^64,65^ leading to a better yield. Ullah et al.^66^ showed strong association of rice grain yield with grain Si contents under stress conditions. These findings strengthen our results on yield improvement under heat stress with application of Si.

Correlation analyses revealed strong positive association of morphological and yield traits with leaf pigments and negative association of antioxidant enzymatic activities and osmoprotectants (Table 1). Mechanism behind is that antioxidant defense mechanism activates in the response of heat stress to scavenge ROS^10,67^ but it compromises physiology and morphological growth^48^ and results in negative correlation. But application of Si improves antioxidant enzymatic activities resulting in more scavenging of ROS and improved yield. Thus, improvement in morphological traits and yield attributed to improvement in leaf pigments as a result of enhanced antioxidant enzymatic activities and more production of osmoprotectants.

## Conclusion

It was concluded that terminal heat stress deteriorates leaf pigments (chlorophyll and carotenoids) due to production of reactive oxygen species which is evident from enhanced antioxidant activities and lead to poor growth and lower yield. Foliaged applied Si at heading stage significantly improved the yield attributes by up regulating the antioxidants defense mechanism and enhanced the levels of osmo-protectants which in turn protected the leaf pigments and compensate the yield losses. Based on overall results, a foliar application of 4 mM Si is suggested to alleviate the adverse effect of terminal heat stress in semi-arid and arid regions.
